# Justified Concerns? An Exploration of the Leg Tuck in a Tactical Population

**DOI:** 10.3390/ijerph192113918

**Published:** 2022-10-26

**Authors:** Robert G. Lockie, Robin M. Orr, J. Jay Dawes

**Affiliations:** 1 Department of Kinesiology, California State University, Fullerton, CA 92831, USA; 2 Tactical Research Unit, Bond University, Robina, QLD 4226, Australia; 3 Tactical Fitness and Nutrition Lab., Oklahoma State University, Stillwater, OK 74078, USA

**Keywords:** abdominal strength, Army Combat Fitness Test, backwards overhead medicine ball throw, between-sex differences, females, firefighters, Illinois agility test, pull-ups, push-ups, tactical

## Abstract

The leg tuck was replaced by the plank in the Army Combat Fitness Test, in part because it was felt it discriminated against women. There is limited leg tuck research, including between-sex comparisons and relationships with other fitness tests. This study investigated the leg tuck in a firefighter trainee population (274 males, 31 females). Archival fitness test data included: Illinois agility test (IAT); push-ups; pull-ups; leg tucks; multistage fitness test; 4.54 kg backwards overhead medicine ball throw (BOMBT); 10-repetition maximum deadlift; and 18 kg kettlebell farmer’s carry over a 91.44 m course. Independent samples *t*-tests (*p* < 0.05) and effect sizes (*d*) compared the sexes. Partial correlations and stepwise regression (controlling for sex; *p* < 0.05) calculated relationships between the leg tuck with the other tests. Male trainees outperformed females in all tests (*p* ≤ 0.003). The largest difference was for the BOMBT (*d* = 2.59) not the leg tuck (*d* = 1.28). The strongest leg tuck relationships were with pull-ups (*r* = 0.790) and push-ups (*r* = 0.553). Sex, pull-ups, and push-ups predicted the leg tuck (*r^2^* = 0.674). Approximately 80% of the females could complete one leg tuck, although female personnel may require specific strength and power training. Pulling strength may be a determining factor in leg tuck performance, which is likely not indicated by the plank.

## 1. Introduction

The leg tuck, or versions of this movement (e.g., hanging knee raises), are unique exercises that are often adopted to target abdominal and hip flexor muscular development [1]. This exercise requires the individual to hang from a pull-up bar with an alternated grip and fully extended arms. The individual then flexes at the elbows, hips, and knees to raise their knees or thighs to their elbows, before returning to the starting position. This exercise anecdotally has been said to stress grip and upper body strength, in addition to abdominal muscular strength and endurance [2]. More recently, the leg tuck was incorporated into the Army Combat Fitness Test (ACFT) for the US Army.

The ACFT was originally designed to measure a soldiers’ readiness for combat, suitability for advancement, and eligibility to remain in the Army, and consisted of six events [2]. These events were selected in part to assess a wide range of physical capacities, including upper and lower body power and strength, core strength, and cardiovascular endurance [2,3]. The events originally included: three-repetition maximum hexagonal bar deadlift; standing power throw (or backwards overhead medicine ball throw; BOMBT) with a 10 lb (4.54 kg) medicine ball; 2 min hand release push-up test; sprint-drag-carry (run up-and-back over a 25 m distance alternating between tasks such as sprinting, dragging a 90 lb (40.82 kg) sled, carrying 40 lb (18.14 kg) kettlebells, and side-shuffling); maximal hanging leg tuck repetitions in 2 min; and 2 mile (3.22 km) run [2,3]. Although the ACFT was introduced in 2019, several challenges were experienced during the rollout [4,5]. A major issue was the higher failure rate of females versus males [4,5,6]. News reports indicated that in the second quarter of 2020, 54% of women failed the ACFT, compared with 7% of men [5]. The leg tuck was identified as one of the major issues that was causing women to fail the ACFT [4,5,6], even though this exercise has been part of US Army doctrine for more than a decade [7,8]. Novak [4] reported that during 6 months of ACFT trials, with approximately 14,000 soldiers, 60% of all female soldiers and 8% of all male soldiers were unable to do one leg tuck, which was the minimum number needed to pass.

In response to criticisms about the inclusion of the leg tuck in the ACFT [4], the US Army recently replaced the leg tuck with the plank [9]. The plank involves an individual lying face down on the ground with their forearms and toes contacting the ground. The individual then raises their hips off the floor to form a straight line from the shoulders to the heels, with a neutral back, holding this position either for a set duration (e.g., 30 s) [10] or for as long as they can [11]. The plank was introduced as a replacement for the leg tuck despite the likelihood that these two exercises measure different physical qualities. Previous research has shown that different exercises designed to target the abdominal region can result in differences in technique and muscle activation [1,12]. Moreover, as reported by Winkie [9], the ACFT is now viewed as a general physical fitness assessment. This would seem counterintuitive given that soldiers need to be physically prepared for combat within their job duties.

There was specific reasoning behind the inclusion of the leg tuck in the ACFT. Information from the US Army suggested that the leg tuck related to common soldiering tasks [13], with examples including surmounting obstacles and walls, and climbing, descending, or traversing ropes [2]. There is some basis supporting these suggestions. As an example, Lockie et al. [14,15] found that the number of sit-ups completed in 60 s correlated (*r* = −0.15 to −0.23, *p* ≤ 0.01) with the ability to perform a chain link fence and solid wall climb in law enforcement recruits. Despite the criticisms of the exercise itself and process of including the leg tuck in the ACFT [4,6], the removal of the leg tuck from the ACFT appears to have been made with minimal analysis of this specific exercise. For instance, limited data has been presented on the specific differences between the sexes in the leg tuck beyond the reporting of failure rates [4]. It would be beneficial to document the magnitude of difference between males and females in the leg tuck relative to other fitness tests, to understand how different the sexes may be. Further, there has been no investigation of relationships between the leg tuck and other fitness tests. An analysis of relationships between the leg tuck with other fitness tests could provide specific insight into what physical qualities should have been developed to improve leg tuck performance. By extension, potential job tasks that could be enhanced by the physical qualities required in the leg tuck (e.g., rope climbing and clearing fences and obstacles) could be identified. It would be beneficial to provide some analysis of the leg tuck to determine whether the changes made by the US Army were appropriate. Furthermore, any other tactical organizations that may use the leg tuck as a test of exercise within training programs should know more about this exercise and potential adaptations that could be experienced by personnel.

Therefore, this study used a sample of convenience in firefighter trainees who completed the leg tuck amongst a battery of other fitness tests. As firefighters require a range of different physical abilities when in the field [16,17,18,19,20], the fitness testing battery featured upper and lower body strength and power tests, in addition to measures of anaerobic and aerobic fitness (similar to the ACFT). Although the job demands and physical capacities of populations such as soldiers [21,22,23] and firefighters [16,17,18,19,20] may vary, it would still be beneficial to provide an analysis of tactical personnel within the context of comparing the sexes and correlating different fitness tests with the leg tuck. Additionally, becoming a firefighter is a career path for many soldiers [24], and soldiers may need to perform firefighting job tasks in the field [20]. This study involved the analysis of archival data, so accordingly the researchers did not have input into the fitness tests selected by the fire department training staff. Nonetheless, the data available was convenient as the training staff incorporated the leg tuck within their fitness testing battery. Further research on the leg tuck has been recommended in the tactical research [6], so it is important to follow-up on these suggestions. It was hypothesized that the male trainees would outperform the female trainees in all fitness tests, with the magnitude of difference being the greatest for the leg tuck. It was further hypothesized that the leg tuck would correlate and be predicted by sex, in addition to tests that require upper body strength and endurance (i.e., pull-ups and push-ups).

## 2. Materials and Methods

### 2.1. Subjects

De-identified archival data from six academy classes from one fire department were released to the researchers for this retrospective investigation. The data sample was comprised of 305 firefighter trainees, which included 274 males and 31 females. Similar to previous first responder research, demographic information was not provided to the researchers [25,26,27]. However, all trainees were above 18 years of age and had completed a pre-placement medical evaluation [28]. The six training cohorts started their academy during 2020 in southern California. The weather conditions across the year were typical of that for southern California [29]. Based on the archival nature of this study, the institutional ethics committee approved the use of pre-existing data (HSR-17-18-401). The study also conformed to the Declaration of Helsinki [30].

### 2.2. Procedures

The fitness tests were administered by training staff at the start of academy within a single, 90 min physical training session [27]. The primary purpose of the fitness test battery was to identify areas in need of improvement within the trainees and was overseen by a Certified Strength and Conditioning Specialist. The use of fitness test data for training prescription is an expectation within the strength and conditioning profession [31], so the tests had to be wide-ranging such that a number of different fitness qualities could be assessed. This was beneficial to the current study as several different tests could then be examined relative to the leg tuck. It should be stated that the fitness tests were not used by training staff to analyze job performance readiness per se. However, the data does provide an overview of the fitness characteristics of firefighter trainees. The tests were completed in the order presented, which was standard practice for the fire department. While there may have been order effects due to the manner in which the tests were conducted [31], the order was consistent across all trainees. Testing occurred outdoors at the fire department’s training facility, and enough time was provided between test attempts to ensure adequate recovery [31].

### 2.3. Illinois Agility Test

**Figure 1:**
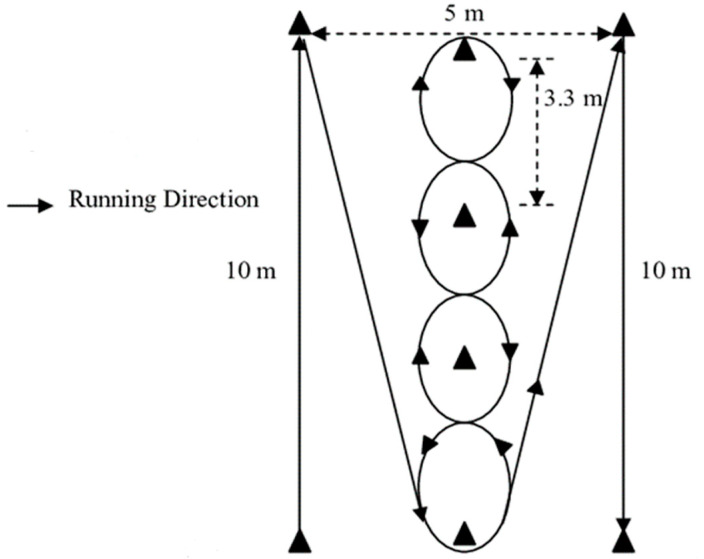
Dimensions and running direction for the Illinois Agility Test.

The Illinois Agility Test (IAT) was used by training staff to measure change-of-direction speed (Figure 1) [32,33], and has been utilized previously in tactical personnel [34,35,36]. This test involved four markers placed to indicate an area that was 10 m long and 5 m wide. In the center area, four markers were placed 3.3 m apart. Trainees began in the prone position behind the start point and outside the first cone. The tester gave a preparatory command of “Ready”, before the command of “Go”. The trainee then jumped to their feet and navigated the course around the cones as quickly as possible. Trainees were instructed not to cut over or contact the markers and to follow the prescribed route throughout the entirety of the trial. If a trainee failed to follow these protocols, or they slipped during the trial, the trial was stopped and re-attempted. Time was recorded in seconds (s) via a stopwatch, from the initiation of movement until the trainee crossed the finish line. Testers trained in the use of stopwatch timing procedures for running tests can record reliable data [37]. Depending on time constraints, 1–2 trials were completed by trainees, with the fastest trial used for analysis.

### 2.4. Metronome Push-Ups

Maximal push-ups provided a measure of upper body muscular endurance [27,38]. Metronome push-ups performed at a cadence of 80 beats per minute were employed to measure upper body muscular endurance. A metronome audio file was played during the test. On the command “Get ready”, trainees were to assume the kneeling push-up position with the arms extended. On the command “Get set”, trainees adopted the standard ‘up’ position (body taut and straight, hands positioned shoulder-width apart, fingers pointed forwards, and knees off the ground) for the push-up [39]. On the command “Go” and metronome initiation, the trainee lowered themselves until their upper arms were parallel to the ground in the ‘bottom’ position. On the next metronome sound, they immediately returned to the ‘up’ position. On the next metronome sound, the trainee immediately lowered to the bottom position, and so forth. The test was terminated when the trainee could no longer complete repetitions in time with the cadence. If the trainee maintained the cadence, but did not meet other standards (i.e., they did not extend the elbows fully, they failed to lower until the upper arms were parallel to the ground, or there was a sag in the pelvis/trunk), the grader repeated the number of the last correct repetition and told the trainee to make the proper correction. The total number of correct repetitions performed was recorded as the final score.

### 2.5. Pull-Ups

The pull-up test provided a measure of upper body pulling strength [40], and has been adopted in previous tactical populations [14,15,41,42,43]. On the command “Get ready”, trainees moved to a free-hang position with their hands positioned shoulder-width apart with a pronated grip, thumbs wrapped around the bar, and elbows extended. On the command of “Go”, the trainee maintained a vertical body alignment and pulled themselves upward until the chin was over the bar to complete one repetition. The trainee then descended to the start position where their arms were fully extended, and they continued to complete repetitions until they could no longer raise their chin over the bar. If the trainee kicked with their lower limbs when raising, the repetition was not counted. The final score was the number of correct repetitions performed.

### 2.6. Leg Tucks

As stated, the leg tuck has been reported to measure grip, arm, shoulder, and trunk muscle strength [2]. On the command, “Get ready”, the trainee moved to a free-hang position with extended arms, and their hands positioned with an alternated grip on the pull-up bar such that their body faced the length of the bar. On the command “Go”, the trainee lifted their lower body upward so that their elbows flexed to approximately 90° while simultaneously tucking their knees to contact their elbows. The knees had to contact the elbows for a repetition to count. The trainee then returned to the hang position and repeated this sequence as many times as possible. The body of the trainee was to be extended in the free-hang position between each repetition, and they could not rest the legs on the bar or swing past the starting position upon lowering. The score was the number of correct repetitions performed.

### 2.7. Estimated Maximal Aerobic Capacity (V̇O_2max_)

Estimated maximal aerobic capacity (V̇O_2max_) was derived from the 20 m multistage fitness test (MSFT) which was conducted according to established procedures [38]. Trainees ran back and forth between two lines indicated by markers spaced 20 m apart. The running speed for this test was standardized by pre-recorded auditory cues (i.e., beeps) played from an audio file. The test was terminated when the trainee was unable to reach the lines twice in a row in accordance with the auditory cues. This test was scored according to the final stage the trainee was able to achieve. V̇O_2max_, measured in milliliters per kg body mass per minute (mL·kg^−1^·min^−1^), was estimated for each trainee based on the table from Ramsbottom et al. [44]. The reader is directed to the work of Ramsbottom et al. [44] to review the conversion chart.

### 2.8. Backwards Overhead Medicine Ball Throw (BOMBT)

The BOMBT with a 4.54 kg (10 lbs) medicine ball was used to assess combined upper and lower body power and coordination [45,46]. The trainee stood with their back to the throwing area, with their feet shoulder-width apart and heels on the start line. The ball was held in front of the body, with the arms extended at shoulder height. In one movement, trainees flexed at the hips, knees and trunk, lowering the ball below the waist. The trainee then extended their legs and thrust the hips forwards, while flexing the shoulders and elevating the ball above shoulder height as they threw it back over their head. Following the throw, the trainees’ feet could leave the ground; however, their body could not go past the start line. Horizontal distance was measured via a tape measure from the start line to the point where the ball first contacted the ground.

### 2.9. 10-Repetition Maximum (10RM) Deadlift

The 10-repetition maximum (10RM) deadlift was used to measure lower body strength by training staff. The deadlift was performed as described in the literature [27], although trainees could self-select their stance foot placement and grip. Trainees performed warm-up sets as needed (up to 10 repetitions) with different loads (52 kg (115 lbs), 74 kg (175 lbs), 84 kg (185 lbs), 102 kg (225 lbs)). After the warm-up sets, the weight was progressively increased, and trainees completed 10 repetitions which were counted by a staff member. A successful repetition occurred when the trainee was standing with their shoulders positioned behind the vertical orientation of the bar via knee extension, their shoulders were retracted, and they held an erect stance. A pause of up to 2 s at the top of the lift was allowed between repetitions. The trainee then lowered the weight to the ground in a controlled manner; no between-repetition rest was allowed when the weight was on the ground. The test was terminated if the trainee did not attain the correct upright position during a repetition, they exceeded the approximate 2 s time limit at the top or bottom of a repetition, they dropped the weight, or they failed to keep the bar ascending during a repetition. Approximately 2–3 min were provided between attempts. The load for the last successful 10RM attempt was recorded in lbs and converted to kg for this study.

### 2.10. Farmer’s Carry

As firefighters perform loaded carries as part of their occupation [17], training staff included a kettlebell farmer’s carry in the test battery. To complete the test, trainees carried 18 kg (40 lb) kettlebells (one in each hand) four times up-and-back over a 22.86 m (25 yards) distance as quickly as possible, which corresponded to a total distance of 91.44 m (100 yards). To begin, the trainee stood at the start line with the kettlebells positioned on the ground on either side of the trainee. On the command “Go”, the trainee squatted down and lifted the two kettlebells and proceeded to cover the 4 × 22.86 m course by walking, jogging, or running. If trainees dropped a kettlebell at any point, they could pick them up and continue. Time was recorded via stopwatch in seconds from the initiation of the movement until the trainee completed the course.

### 2.11. Statistical Analysis

Statistical analyses were computed using the Statistics Package for Social Sciences (Version 28.0; IBM Corporation, New York, NY, USA) and Microsoft Excel (Microsoft Corporation^TM^, Redmond, WA, USA). Descriptive statistics (mean ± standard deviation [SD]) were calculated for each test parameter. Independent samples *t*-tests calculated any differences in fitness test performance between the male and female trainees. Levene’s test for equality of variances ascertained the homogeneity of variance for the data. The level of significance was set at *p* < 0.01 due to the number *t*-tests performed and to limit family-wise error [38]. Effect sizes (Cohen’s *d*; difference between the means divided by the pooled standard deviations) were used to derive the magnitude of difference between the sexes in the fitness tests [47]. A *d* less than 0.2 was a trivial effect; 0.2 to 0.6 a small effect; 0.6 to 1.2 a moderate effect; 1.2 to 2.0 a large effect; 2.0 to 4.0 a very large effect; and 4.0 and above an extremely large effect [48].

Partial correlations controlling for sex were used to investigate relationships between the leg tuck and the other fitness tests (IAT, push-ups, pull-ups, estimated V̇O_2max_, BOMBT, 10RM deadlift, and farmer’s carry). Significance was set at *p* < 0.05. The strength of the relationships were defined as: an *r* between 0 to ±0.3 was small; ±0.31 to ±0.49, moderate; ±0.5 to ±0.69, large; ±0.7 to ±0.89, very large; and ±0.9 to ±1 near perfect for relationship prediction [49]. Stepwise linear regression analyses (*p* < 0.05), with sex as a control variable, was used to determine whether any of the fitness tests predicted leg tuck performance in the trainees.

## 3. Results

Descriptive data for all trainees combined and by sex are shown in Table 1. Equal variances were assumed for all variables except the 10RM deadlift. Male trainees were significantly superior to the female trainees in all fitness tests. There was a very large effect for the BOMBT between-sex difference and large effects for the differences between pull-ups, leg tucks, and the 10RM deadlift. Moderate effects were seen for the between-sex differences in the IAT, estimated V̇O_2max_, and farmer’s carry, and a small effect for push-ups. Six out of 274 males (2.2% of the sample) could not do a pull-up, while one male (0.4% of the sample) could not do a leg tuck. For the females, six out of 31 (19.4% of the sample) could not do a pull-up or leg tuck. This also meant that 25 females (80.6% of the sample) could perform at least one pull-up or leg tuck.

The leg tuck correlation data is shown in Table 2. There was a very large, positive relationship with pull-ups. A large, positive relationship existed with push-ups, and a moderate relationship with estimated V̇O_2max_. Each of these relationships indicated that a greater number of leg tuck repetitions related to either a higher number of pull-up or push-up repetitions, or a higher estimated V̇O_2max_. There were small, negative, relationships between leg tucks and the IAT and 10RM deadlift. These relationships suggested that a higher number of leg tuck repetitions related to a faster IAT, but also a lower 10RM deadlift. The leg tuck did not significantly correlate with the BOMBT or farmer’s carry.

**Figure 2:**
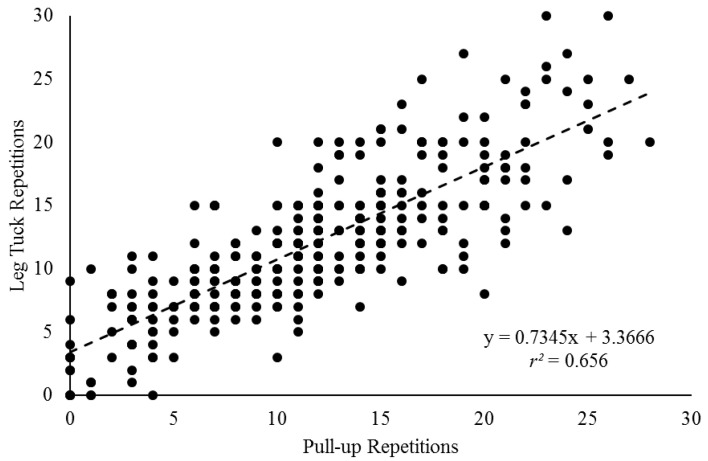
Regression scatter plot for firefighter trainees (*N* = 305) between leg tuck and pull-up repetitions.

The stepwise regression data is displayed in Table 3. Sex explained 12.1% of the variance in leg tuck performance. When pull-ups were added to the equation, 66.8% of the variance was explained. Push-ups slightly, albeit significantly, increased the explained variance to 67.4%. A regression scatter plot was produced for the relationship between leg tucks and pull-ups, and this can be viewed in Figure 2. The explained variance was approximately 65.6%.

## 4. Discussion

The US Army recently removed the leg tuck from the ACFT [9] following criticisms regarding how it negatively impacted female soldiers [4]. Nonetheless, there has been little specific analysis of between-sex differences in the leg tuck beyond describing failure rates [4]. Further, there has been no analysis of the relationships between the leg tuck and other fitness tests to ascertain what physical qualities might be important for this exercise. This study investigated the leg tuck in a tactical population of firefighters, which was a sample of convenience as they had completed the leg tuck as part of a general fitness testing battery. In support of the study hypotheses, male firefighter trainees outperformed female trainees in all fitness tests, including the leg tuck. However, the test with the largest magnitude of difference between the sexes was the BOMBT (*d* = 2.59) and not the leg tuck (*d* = 1.28). There were several significant relationships between the leg tuck with the other fitness tests (IAT, push-ups, pull-ups, estimated V̇O_2max_, and 10RM deadlift), and sex, pull-ups, and push-ups predicted leg tuck performance. In particular, the ability to do pull-ups related to leg tuck performance. These results have implications for tactical populations, including the US Army. Although further analysis is needed, it is likely that physical qualities considered important for the leg tuck are not being captured by the plank. Furthermore, targeting upper body pulling strength could aid leg tuck performance, and job tasks that are potentially indicated by the leg tuck (e.g., climbing and traversing ropes, clearing obstacles).

Numerous studies in tactical populations have documented the tendency for males, in general, to outperform females in a range of different fitness tests [38,50,51,52,53,54]. This was also the case in the current study. Men, in general, tend to be physically larger [55] and have more muscle mass [56], which likely contributed to the superior performance of the male trainees in this study for all fitness tests, including the leg tuck. Interestingly, despite the failure rates and high numbers of females in the military who could not perform the leg tuck [4,5], the greatest magnitude of difference was not observed for this test. Rather, it was for the BOMBT, which assessed combined upper and lower body power and coordination [45,46]. Fitness tests and occupational job tasks that require maximal power tend to be challenging for females when compared to males [54,57,58]. This is not to say females cannot complete physically demanding jobs in firefighting, military, or law enforcement; rather, it highlights the physical challenges many females will encounter. Previous research often recommends specific strength and power training for female tactical populations, and the results from this study support these assertions [54,57]. Nevertheless, in this sample of female firefighter trainees, the most challenging fitness test did not appear to be the leg tuck.

As previously stated, 80.6% of the females in this sample (*n* = 25) could complete at least one leg tuck. However, this also meant 19.4% of female trainees (*n* = 6) could not complete one leg tuck. Novak [4] described adverse impacts on female soldiers completing the leg tuck, which may translate to unintentional discrimination. As described by Novak [4], adverse impacts can occur if the selection rate for a certain group is less than 80% of that of the group with the highest selection rate. It should be clearly stated that this fire department did not use these fitness tests as a measure of job readiness, so any indication of adverse impact for any of the fitness tests presented in this study is not applicable. Nonetheless, it is interesting that within the sample of females (albeit small), just under 20% were not able to do one leg tuck. A further note is that this percentage of females was substantially less than the reported percentage of female soldiers from the US Army (approximately 60% of 14,000) who could not perform a single leg tuck [4]. Obviously, the sample size of females from this study is lower than that reported by Novak [4]. Nonetheless, most females in this study could still perform at least one leg tuck. Firefighter trainees require some level of physical conditioning as they must complete the Candidate Physical Ability Test (CPAT) prior to being accepted to a training academy [17]. The CPAT simulates job tasks (stair climb, hose drag, equipment carry, ladder raise and extension, forcible entry, search, rescue drag, and ceiling breach and pull) to measure a candidate’s ability to perform the physically demanding tasks of firefighting [59]. It could be surmised from this study that fitter females should be able to perform a leg tuck. For the US Army, physical fitness development of their female students may have been of greater impact on leg tuck performance than sex alone. A larger potential issue is that by using the plank, soldiers lacking important physical qualities may still be considered fit enough based upon their ACFT results. Specific to the leg tuck, this concept should be examined further using correlations with other fitness tests.

Several of the fitness tests used by this fire department correlated with the leg tuck, including pull-ups, push-ups, estimated V̇O_2max_, IAT, and 10RM deadlift. The pull-ups, push-ups, estimated V̇O_2max_, and IAT relationships indicated that greater leg tuck repetitions related to better performance in each test. This may be indicative of findings shown in law enforcement research, where fitter individuals tend to perform well in all fitness tests regardless of the primary quality that is assessed (e.g., muscular strength, power, and endurance, anaerobic and aerobic fitness) [14,15,53]. The relationships with pull-ups and push-ups were noteworthy given the strength of their correlation (very large and large, respectively) and the fact they also predicted leg tuck performance. Pull-ups measure upper body pulling strength [40], while push-ups provide a metric for upper body muscular endurance [38]. Both qualities are likely required to perform multiple leg tuck repetitions. Additionally, the US Army linked the leg tuck with soldiering tasks such as the ability to climb ropes and clear obstacles [2,13]. In law enforcement recruits, greater pull-up and push-up repetitions have been associated with faster chain link fence and solid wall climbs [14,15]. If the results from this study provided similar results in soldiers, the leg tuck could have provided an indication of pulling and pushing ability in military personnel. It is doubtful whether the plank would provide similar information as part of the ACFT. However, the current study cannot address whether the plank relates to other fitness qualities such as that detailed for the leg tuck. Further research is needed to specifically answer this question.

As has been discussed, a major criticism of the leg tuck within the ACFT was that it unfairly penalized female soldiers [4]. Within the regression analyses for the current research, sex explained 12.1% of the variance with leg tucks. However, when pull-ups were added to the equation the explained variance jumped to 66.8% (Table 3). When considering just the pull-ups and leg tucks without the influence of sex, the explained variance was approximately 66% (Figure 2). Although there was not another specific abdominal test feature in the testing battery from this fire department, limitations in performing the leg tuck may not be abdominal strength. Focusing on the leg tuck as a test of abdominal muscular endurance may not have been appropriate. Rather, upper body pulling strength may be a more important consideration. By selecting an exercise such as the plank to replace the leg tuck, the US Army may be missing the actual limitations (i.e., upper body pulling strength) in their female personnel, in addition to male personnel who struggle with the leg tuck. This is not to say that abdominal strength and endurance is not important for soldiers. However, if a soldier is lacking in upper body pulling (and pushing) strength, this could disadvantage them in tasks where these qualities could be essential (e.g., climbing ropes, scaling obstacles and fences, hand-to-hand combat). For any tactical organizations who use the leg tuck in testing or training, it would be important to recognize that upper body pulling and pushing strength-endurance are the physical qualities that are predominantly impacted.

Despite the application of the results of the current research, it should be acknowledged that this study did not involve a direct analysis of the leg tuck as part of the ACFT. Instead, a sample of convenience of firefighter trainees was used that had available leg tuck data. Military personnel may display different results to the firefighter trainees detailed in this study. Nonetheless, the important physical qualities that contribute to the leg tuck would likely be similar across tactical personnel, and this approach has been used in other research that has used surrogate populations (e.g., civilians) when analyzing tactical fitness tests or job tasks [32,60,61]. Furthermore, some soldiers also work as firefighters [20,24], and could have to perform firefighting job tasks in the field [20]. The fitness tests used for the correlation analysis were also selected via convenience as they were part of the battery adopted within this fire department. Accordingly, there was not another abdominal exercise (e.g., crunches, sit-ups, or the plank) that was involved in the between-sex, correlation, or regression analyses. Indeed, inclusion of the plank in this study would have been beneficial considering it has replaced the leg tuck in the ACFT [9]. Future research should investigate how much an abdominal muscular strength or endurance test such as the plank, or other potential options such as sit-ups [14,15,29,38,52,53,62] or curl-ups [63], relates to the leg tuck and other fitness tests. Nonetheless, it is plausible that the ability to perform pull-ups and push-ups would still have stronger relationships with the leg tuck.

There are other study limitations that should be identified. Retrospective data was analyzed in this study. Accordingly, and as stated, the researchers did not have input into what tests were conducted and what procedures were used. This limited the scope of the current study. Although this has occurred in other first responder research [25,26,27], descriptive data about the subjects were not provided to the researchers. This may limit how the current data could be compared across other tactical studies. There was a discrepancy between the sexes in this study (male *n* = 274; female *n* = 31). However, this is very common in tactical research analyzing physically demanding occupations [14,15,17,27,29,34,38,52,53,54,62], and almost impossible to avoid in research. Regardless, given how many fire departments are actively trying to recruit and retain more women [64], the presentation of any data for female firefighters has larger benefits within tactical research. The testing order did not follow the recommendations of the National Strength and Conditioning Association [31], and this could have affected the data that was collected. Nonetheless, the testing order was standard practice for this department and was consistent for all trainees, and sufficient recovery periods were provided between tests. Body mass and composition would also likely affect leg tuck performance [62,65], and this should be analyzed in future studies. The sample size of firefighters in this study was much lower than the number of soldiers reported by Novak [4]. Nonetheless, given the current lack of research investigating the leg tuck, this study provided an important contribution to the literature.

## 5. Conclusions

Male trainees outperformed female trainees in all the fitness tests, including the leg tuck. The greatest magnitude of difference was not for the leg tuck but rather the BOMBT, and most females in this sample were able to perform at least one leg tuck. The leg tuck correlated with several fitness tests in firefighter trainees, including pull-ups, push-ups, estimated V̇O_2max_, and the IAT. Pull-ups and push-ups also predicted the leg tuck. Upper body pulling strength particularly appeared to be an important contributor to the completion of leg tucks, potentially more so than abdominal strength. Initially, the current data suggested female firefighter trainees likely need specific strength and power training to optimize fitness test and job performance. In addition to this, the current study provided some data that may question whether the plank is the best substitute for the leg tuck in the ACFT (although this requires further specific analysis in Army personnel), and whether its inclusion may divert from identifying those at need of further upper body conditioning. This is because soldiers lacking in upper body pulling strength and muscular endurance may still be able to perform the plank. Even though the ACFT may have shifted towards a more general fitness assessment, if results from this test are used to drive a soldier’s training this could be an issue as training staff may not receive a full picture of an individual’s strengths and limitations. Indeed, if a soldier is lacking in upper body pulling and pushing strength, this could disadvantage them in tasks such as climbing ropes, scaling obstacles, and hand-to-hand combat. More research is required to document relationships between the leg tuck and plank in tactical personnel, any other appropriate substitutions for the leg tuck or plank, the influence of these relationships, and the longer-term impacts of revising the standards within the ACFT.

## Figures and Tables

**Table 1:** Descriptive data (mean ± SD) for fitness test performance (Illinois agility test [IAT], metronome push-ups, pull-ups, 4.54 kg backwards overhead medicine ball throw [BOMBT], leg tuck, estimated maximal aerobic capacity [V̇O_2max_] from the 20 m multistage fitness test, 10RM deadlift, and the farmer’s carry) for firefighter trainees.

Tests	Overall (*N* = 305)	Males (*n* = 274)	Females (*n* = 31)	*p*	*d*	*d* Strength
IAT (s)	18.44 ± 1.42	18.31 ± 1.40	19.60 ± 1.00 *	<0.001	1.06	Moderate
Push-ups (no.)	61.88 ± 23.08	63.20 ± 22.73	50.32 ± 23.28 *	0.003	0.56	Small
Pull-ups (no.)	11.70 ± 6.39	12.45 ± 6.13	5.10 ± 4.68 *	<0.001	1.35	Large
BOMBT (m)	9.53 ± 1.71	9.87 ± 1.41	6.56 ± 1.13 *	<0.001	2.59	Very Large
Leg Tuck (no.)	11.95 ± 5.81	12.64 ± 5.49	5.90 ± 5.01 *	<0.001	1.28	Large
Estimated V̇O_2max_ (mL·kg^−1^·min^−^^1^)	46.00 ± 5.90	46.49 ± 5.80	41.61 ± 4.87 *	<0.001	0.91	Moderate
10RM Deadlift (kg)	143.53 ± 15.17	145.75 ± 12.74	123.72 ± 20.24 *	<0.001	1.30	Large
Farmer’s Carry (s)	28.90 ± 4.15	28.46 ± 4.03	32.67 ± 3.13 *	<0.001	1.17	Moderate

* Significantly (*p* < 0.05) different from the male firefighter trainees.

**Table 2:** Descriptive data (mean ± SD) for fitness test performance (Illinois agility test [IAT], metronome push-ups, pull-ups, 4.54 kg backwards overhead medicine ball throw [BOMBT], leg tuck, estimated maximal aerobic capacity [V̇O_2max_] from the 20 m multistage fitness test, 10RM deadlift, and the farmer’s carry) for firefighter trainees.

Test	Leg Tuck	
	*r*	*p*
IAT	−0.267 *	<0.001
Push-ups	0.553 *	<0.001
Pull-ups	0.790 *	<0.001
BOMBT	0.070	0.231
Estimated V̇O_2max_	0.465 *	<0.001
10RM Deadlift	−0.198 *	<0.001
Farmer’s Carry	0.053	0.368

* Significant (*p* < 0.05) relationship with the leg tuck.

**Table 3:** Stepwise linear regression analysis for the leg tuck with sex and the other fitness tests (Illinois agility test, metronome push-ups, pull-ups, 4.54 kg backwards overhead medicine ball throw, estimated maximal aerobic capacity from the 20 m multistage fitness test, 10RM deadlift, and the farmer’s carry) for firefighter trainees (*N* = 305). Significance was *p* < 0.001.

Variables	*r*	*r* ^2^	Adjusted *r*^2^
Sex	0.353	0.124	0.121
Sex, Pull-ups	0.819	0.668	0.668
Sex, Pull-ups, Push-ups	0.823	0.674	0.674

## Data Availability

Restrictions apply to the availability of these data due to ethical, legal and privacy concerns.
